# Tissue fibrocytes in patients with mild asthma: A possible link to thickness of reticular basement membrane?

**DOI:** 10.1186/1465-9921-7-50

**Published:** 2006-03-29

**Authors:** Kristian Nihlberg, Kristoffer Larsen, Anna Hultgårdh-Nilsson, Anders Malmström, Leif Bjermer, Gunilla Westergren-Thorsson

**Affiliations:** 1 Department of Experimental Medical Science, Section for Vascular and Airway Research, Lund University BMC C13, S-221 84 Lund, Sweden; 2 Department of Respiratory Medicine and Allergology, Lund University Hospital, S-221 85 Lund, Sweden

## Abstract

**Background:**

Myofibroblasts, proposed as being derived from circulating fibrocytes, are considered to be important cells in thickening of the basement membrane in patients with asthma. We have studied the correlation of tissue fibrocyte levels to basement membrane thickness and the presence of fibrocytes in bronchoalveolar lavage fluid (BALF) in steroid-naive patients with mild asthma and controls.

**Methods:**

Patients with mild asthma (n = 9) were recruited and divided into two categories based on whether or not fibroblast-like cells could be established from BALF. Non-asthmatic healthy subjects (n = 5) were used as controls. Colocalization of the fibrocyte markers CD34, CD45RO, procollagen I, and α-smooth muscle actin (α-SMA) were identified in bronchial biopsies from patients and controls by confocal microscopy. Kruskall-Wallis method was used to calculate statistical significance and Spearman coefficient of rank correlation was used to assess the degree of association.

**Results:**

In patients with BALF fibroblasts, a 14-fold increase of tissue cells expressing CD34/CD45RO/α-SMA and a 16-fold increase of tissue cells expressing CD34/procollagen I was observed when compared to controls (p < 0.05). In contrast, patients without BALF fibroblasts displayed a 2-fold increase when compared to controls (p < 0.05). Fibrocytes were localized close to the basement membrane which was significantly thicker in patients with BALF fibroblasts when compared to the other two groups of subjects. Furthermore, basement membrane thickness could be correlated to the number of fibrocytes in tissue (r = 0.711). Fibroblasts-like cells were cultured from BALF where 17.6% of these cells expressed CD34, CD45RO and α-SMA.

**Conclusion:**

These findings indicate a correlation between recruited fibrocytes in tissue and thickness of basement membrane. Fibroblast progenitor cells may therefore be important in airway remodeling in steroid-naive patients with mild asthma.

## Background

Asthma is characterized by airway inflammation and structural remodeling of the lung [1]. These processes may result in sub-epithelial fibrosis that leads to thickening of the lamina reticularis, which contains an increased amount of connective tissue components such as collagens, tenascin, fibronectin and proteoglycans [2-4]. Fibroblasts are considered to be important key cells in this mechanism since they migrate to sites of injury where they acquire a myofibroblast phenotype, upon activation by factors such as TGF-β1, which is followed by production of a new extracellular matrix [5,6]. However, considering the importance of fibroblasts in airway remodeling it is important to establish the possible origins of these cells which today are unclear. It has been postulated in several in vivo models and in patients with allergen-induced asthma that lung tissue repair and regeneration after injury involve the selective recruitment of circulating fibroblast progenitor cells, termed fibrocytes [7-9]. These cells comprise a minor component (<1%) of the circulating pool of leukocytes and have been characterized as antigen presenting cells, which express a specific pattern of both mesenchymal and hematopoetic markers including procollagen I, alpha-smooth muscle actin (α-SMA), CXCR4, CD34, and CD45RO [10]. Fibrocytes have been shown to be important in the generation of fibrosis in several in vivo models where inhibition of fibrocyte recruitment leads to a decrease of fibrotic tissue [11-13]. Furthermore, TGF-β1 is an important inducer of fibrosis and may in addition stimulate fibroblasts in tissue and induce the differentiation of fibrocytes into myofibroblast-like cells with elevated production of extracellular matrix components [14,15]. Although these findings suggest that fibrocytes are important key cells in airway remodeling in patients with allergen-induced asthma and in vivo models, fibrocytes have not been studied in steroid-naive patients with mild asthma.

We have previously shown that activated fibroblasts-like cells may be cultured from bronchoalveolar lavage fluid (BALF) from a sub-group of patients with mild asthma accompanied by elevated levels of eosinophils [16]. These BALF fibroblasts could originate from circulating progenitor cells, such as fibrocytes, which may differentiate into myofibroblast-like cells. We hypothesized therefore if fibrocytes were present in remodeled lung tissue and BALF from steroid-naive patients with mild asthma and, if so, correlated with a thickened basement membrane.

## Methods

### Subjects, bronchoalveolar lavage and sampling of lung tissue

Patients (n = 9) suffering from mild asthma and definite bronchial hyperresponsiveness were included in the study, reviewed and approved by the Swedish Research Ethical Committee (LU339-00). These patients were 25–31 years of age with positive phadiotope staining, non-smokers, PD_20 _< 2 mg/ml of methacholine stimulation, displayed stable mild persistent asthmatic conditions with normal spirometry baseline, free of infections six weeks before bronchoscopy, and had no corticosteroid treatment for six months prior to the study. All of the patients with asthma were atopic and sensitive to pets (cat allergy). Five of the patients with asthma had perennial allergy and one patient had seasonal allergy only (birch pollen). The patients with asthma were further divided into two groups based on whether they displayed the previously described BALF fibroblasts or not (n = 5 and 4, respectively).

The control subjects used (n = 5) were 24–51 years of age. These subjects were non-asthmatic healthy volunteers with a reversibility in FEV_1 _< 12%, with no allergic symptoms, and who did not respond to doses of methacholine lower than 2 mg/ml. BAL was performed by instillation of buffered saline solution with a recovery of more than 70%. Bronchial biopsies were taken from the central airways of the right lung and collected as previously described [17]. Five biopsies were taken from each subject. The biopsies collected did not show any differences in size, vascularity, or muscle content. From each individual 16 sections were analyzed.

### Cell cultures and cytospin analysis

Primary fibroblasts-like cells were established from BALF as previously described [18]. Briefly, BALF was centrifuged at 500 g for 10 min and the supernatant was discarded and the precipitated BALF cells were washed with DMEM. The precipitated cells were cultured in DMEM supplemented with 10% Fetal Clone III (Hyclone, UT), 1% L-glutamine, 0.5% gentamicin and 5 μg/ml amfoterracin, until outgrowth of fibroblast-like cells reached confluence (20–30 days). These fibroblast-like cells were characterized by expression of α-SMA, collagen, and high production of proteoglycans (16). The cultures were trypsinized and used in passage 4–7. Cytospin was performed using a cytocentrifuge (Shandon Southern Products Ltd, Runcorn, Cheshire, UK) where cells in the BALF were collected. The cells were stained using the May-Grünwald/Giemsa (Sigma Aldrich, Stockholm, Sweden) method according to instructions from the manufacturer. 200 cells were counted and granulocytes were identified according to their morphological structure.

### Histological studies and measurement of basement membrane thickness

Bronchial biopsies were fixed in 4% paraformaldehyd, embedded in paraffin and sectioned (5 μm). Basic morphological characterization was made using Gomoris Trichrome staining kit (Polysciences Inc, Warrington, PA) for staining of nuclei, muscles fibers and collagen I. Photographs were taken using a light microscope camera (DM IRB, Leica, Wetzlar, Germany).

Basement membrane thickness was quantified by measuring the area of the entire basement membrane, from the base of the bronchial epithelium to the outer limit of the lamina reticularis, and the length of the true basement membrane. This was performed using a computerized image (Leica Software Q500IW, Leica, Wetzlar, Germany).

Thickness was calculated as area/length of the basement membrane. These measurements were performed as previously described [19].

### Confocal microscopy

Embedded and fixed tissue sections (5 μm) and cultured BALF fibroblasts were dehydrated and incubated with primary monoclonal mouse anti-human antibodies: CD34, CD45RO (BD Biosciences, Pharmingen, Leiden, The Netherlands) and α-SMA with Cy3 conjugates (Sigma Aldrich, Stockholm, Sweden). For cultured BALF fibroblast, cells were fixed in 4% paraformaldehyde and 0.5% Triton X-100 in PBS was used as a permeabilization agent prior α-SMA staining. Secondary antibodies used for detection of CD34 and CD45RO were Alexa Fluor 488 and 647 (Molecular Probes, Eugene, OR), respectively. CD45RO and prolyl-4-hydroxylase detection; fixed tissue sections were dehydrated, treated with 1% trypsin, covered with blocking solution (PBS containing 3%BSA, 5% fatfree milkpowder, and 1% goat serum) and incubated with primary monoclonal antibody CD45RO (BD Biosciences, Pharmingen, Leiden, The Netherlands), prolyl-4-hydroxylase (Dakocytomation, Denmark) and nucleic staining (Sytox Blue Nucleic Acid Stain, Molecular Probes, Eugene, OR). Sections were then washed with TBS+0.5% Triton X. Secondary antibodies used for detection of CD45RO and prolyl-4-hydroxylase were Alexa Flour 488 and 647 (Molecular Probes, Eugene, OR), respectively. For CD34 and procollagen I detection; fixed tissue sections were dehydrated, treated with 1% trypsin, covered with blocking solution (PBS containing 3%BSA, 5% fat-free milk powder, and 1% goat serum) and incubated with primary antibody mouse anti-human CD34 (BD Biosciences, Pharmingen, Leiden, The Netherlands), rat anti-human procollagen I (Chemicon, Europe Ltd, Hampshire, United Kingdom and nucleic staining (Sytox Blue Nucleic Acid Stain, Molecular Probes, Eugene, OR). Sections were then wash with TBS+0.5% Triton X and blocked with 10% goat serum (Vector Laboratories, Burlingame, CA, USA) in PBS. Secondary antibodies used for detection of CD34 and procollagen I were Alexa Flour 488 and 633 (Molecular Probes, Eugene, OR), respectively. Control experiments were performed with/without primary antibody or with/without secondary antibody. Control sections were included in all experiments to correct for background fluorescence. Isotype controls were used in combination and for each fluorochrome to decrease the risk of detecting unspecific staining. Fibrocytes were counted in a subepithelial zone 0–210 μm deep into the reticular zone and divided into area A, B and C (each area 70 μm deep). Results were expressed as the number of cells per 1 mm^2^. All sections with an intact basement membrane and a minimum requirement of intact tissue 70 μm into the reticular zone were counted. Sections were analyzed with Leica confocal-scanning equipment TCS SP2 II (Leica, Wetzlar, Germany).

### Quantification of fibrocytes in cultured BALF fibroblasts

BALF fibroblasts (5000 cells) were cultured 48 h in 4 well chambers. Thereafter 200 cells were randomly choosed for cell counting for their different markers for fibrocytes differentiation.

### Statistical analysis

Mean values ± standard errors of the mean (SEM) were calculated and Kruskall-Wallis was used for analyses of statistical significance (SPSS v7.0, Chicago, IL). All values of p < 0.05 were considered significant. Spearman coefficient of rank correlation was used to assess the degree of association between basement membrane thickness and number of fibrocytes.

## Results

### Tissue fibrocytes are localized in proximity to the basement membrane in patients with mild asthma

**Figure 1:**
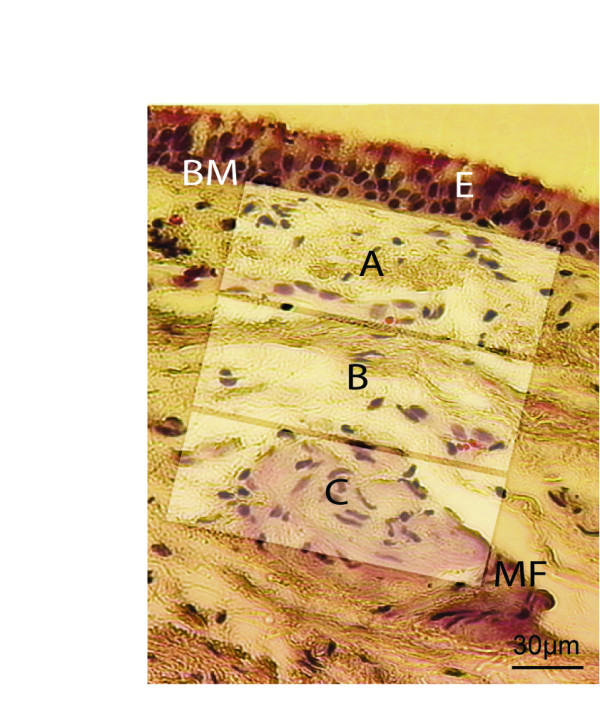
Criteria used when counting fibrocytes in bronchial biopsies. Sections from patients and controls were based on an intact basement membrane together with intact tissue of at least 70 μm in every section. Area A starts were the basement membrane ends. A = 0–70 μm in the section. B = 71–140 μm in the section and C = 141–210 μm in the tissue. *Definition of abbreviations*: BM = Basement membrane, E = epithelium, MF = Muscle fibers.

**Figure 2:**
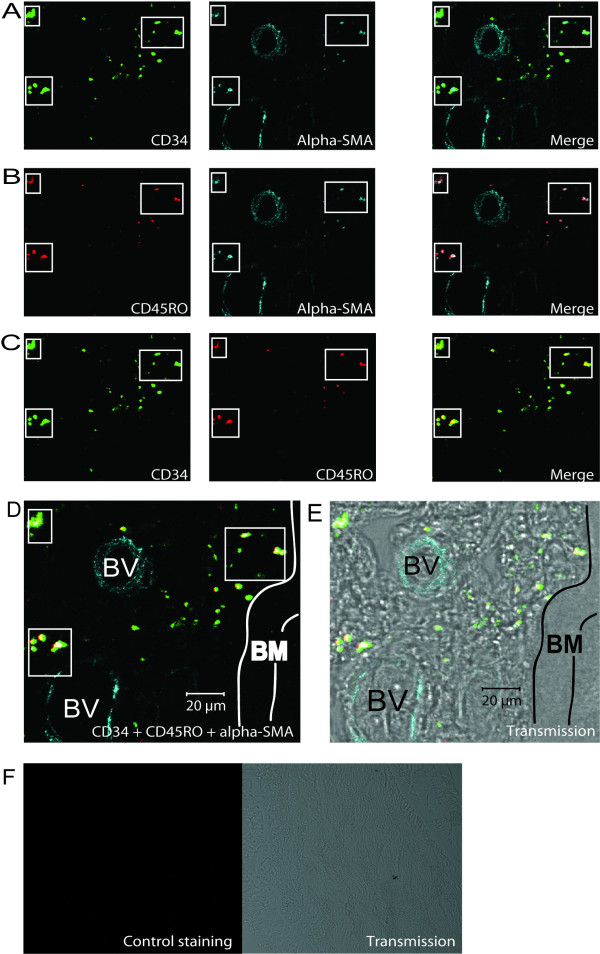
Fibrocytes are localized in proximity to the basement membrane in patients with mild asthma. Bronchial biopsies were stained using immunofluorescent antibodies against CD34(green), α-SMA(blue), CD45RO(red), and were subjected to confocal microscopy. Cells coexpressing CD34/α-SMA (A), CD45RO/α-SMA (B), and CD34/CD45RO (C) is presented where the merged pictures show double positive stained cells. Squares indicate the same area in one tissue section of double and triple positive cells close to the basement membrane. A merged picture illustrating the expression of all antibodies is presented (D) and a transmission picture shows localization of stained cells in tissue (E). Control staining indicates insignificant background staining in confocal and transmission mode (G). *Definition of abbreviations*: BM = Basement membrane, BV = Blood vessel, E = Epithelium.

The sub-epithelial bronchial tissues were divided into three sections (A, B, and C) when studying the localization of fibrocytes (Figure 1). The presence of double stained cells could be analyzed by using antibodies for CD34/α-SMA (Figure 2A), CD45RO/α-SMA (Figure 2B), CD34/CD45RO (Figure 2C), and analyzed by confocal microscope. The indication of a fibrocytes phenotype could be demonstrated by colocalization of all these three markers (Figure 2D). Fibrocytes were primarily localized individually and in clusters close to the epithelium and to blood vessels (Figure 2E). Limited background staining was seen throughout the confocal analysis, indicating specificity for the antibodies used (Figure 2F). Single positive cells were also detected close to triple positive cells in the basement membrane proximity.

**Figure 3:**
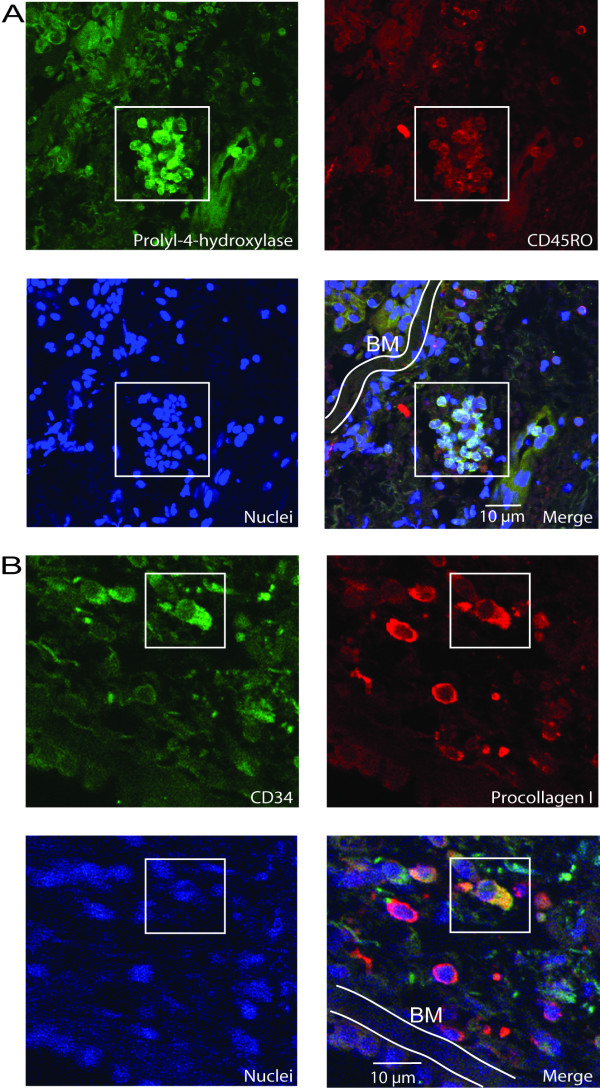
Fibrocytes in patients with mild asthma express collagen. Bronchial biopsies were stained using immunofluorescent antibodies against prolyl-4-hydroxylase (green) CD45RO (red) and nuclei (blue) (A). Squares indicate the same cluster area as shown in A. The merged picture describes the positive cells within the square. Cells were further stained for CD34 (green), procollagen I (red), and nuclei (blue) (B), and were subjected to confocal microscopy. The merged picture describes the positive cell within the square. *Definition of abbreviations*: BM = Basement membrane

Apart from expressing CD34/CD45RO, fibrocytes are further characterized as being collagen producing cells. The cells were therefore stained for prolyl-4-hydroxylase, a protein critically involved in collagen synthesis, and procollagen I. Prolyl-4-hydroxylase/CD45RO could be seen in separate cells and in clusters of cells (Figure 3A). These two markers were also colocalized as shown by a merged picture. The cells did also stain procollagen I/CD34 and the merged picture indicate coexpression of these markers (Figure 3B). Collectively, the merged pictures illustrate positive cells for these markers suggesting an active collagen production in these fibrocytes.

**Figure 4:**
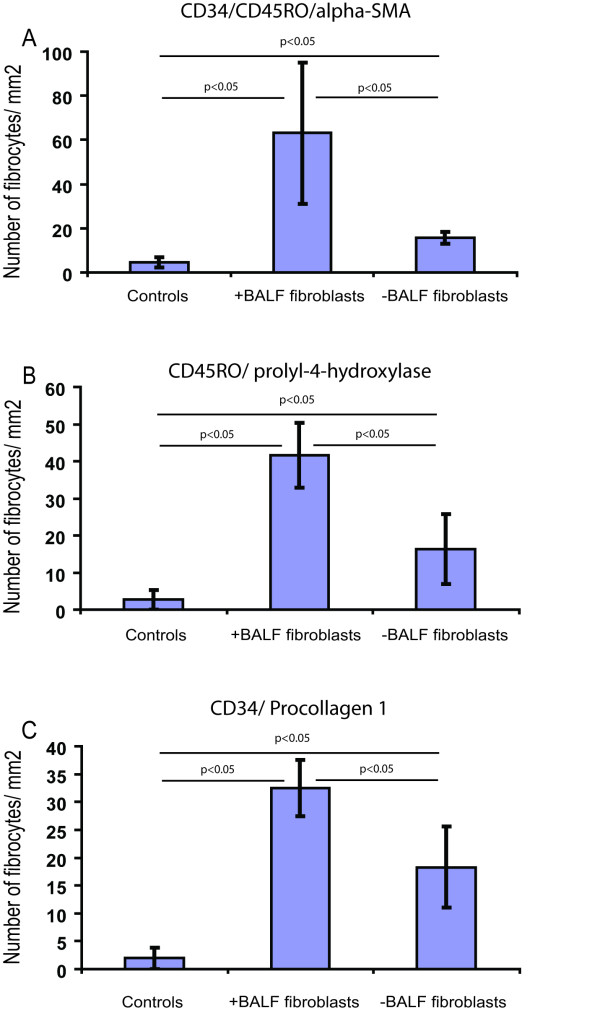
Quantification of CD34, CD45RO, α-SMA, procollagen I and prolyl-4-hydroxylase coexpression in fibrocytes. Cells in bronchial biopsies coexpressing CD34/CD45RO/α-SMA (A), CD45RO/prolyl-4-hydroxylase (B), and CD34/procollagen I (C), were counted. These cells were counted in the areas A and B and data included are from controls, patient with mild asthma with (+) and without (-) BALF fibroblasts and are presented as positive cells/mm^2 ^of tissue section. Values are presented as means ± SEM, n = 4–5 patients.

When counting number of fibrocytes in the tissue, patients with BALF fibroblasts displayed a 14-fold increase (p < 0.01) in the total number of triple positive CD34/CD45RO/α-SMA fibrocytes present in area A + B when compared to controls (Figure 4A). The other patient group without fibroblasts in BALF displayed a significant 2-fold increase (p < 0.05) in fibrocytes when compared to controls. The localization of triple positive cells strongly suggests a gradient of fibrocytes present throughout these areas. In area A, patients with BALF fibroblasts displayed an 11-fold increase of fibrocytes (p < 0.05) when compared to controls (52 ± 32 vs. 5 ± 2) and a 5-fold increase when compared to patients without BALF fibroblasts (52 ± 32 vs. 12 ± 1). This pattern was similar in area B where fibrocytes were observed in patients with and without BALF fibroblasts (11 ± 6 vs. 4 ± 2) but not in the controls. In area C, fibrocytes were only detected in patients with BALF fibroblasts (3 ± 1) and controls (1 ± 1). When quantifying the number of prolyl-4-hydroxylase/CD45RO positive cells, patients with BALF fibroblasts showed 2.5-fold elevated levels of these cells when compared to the other patient group (p < 0.05) and 17-fold increase when compared to controls (p < 0.05) (Figure 4B). A similar pattern was seen when quantifying CD34/procollagen I positive cells (Figure 4C), where a 2-fold increase (p < 0.05) in double positive cells were seen in patients with BALF fibroblasts when compared to the other patient group and a 16-fold increase when compared to controls (p < 0.05).

### Fibrocytes levels correlate to thickness of basement membrane

**Figure 5:**
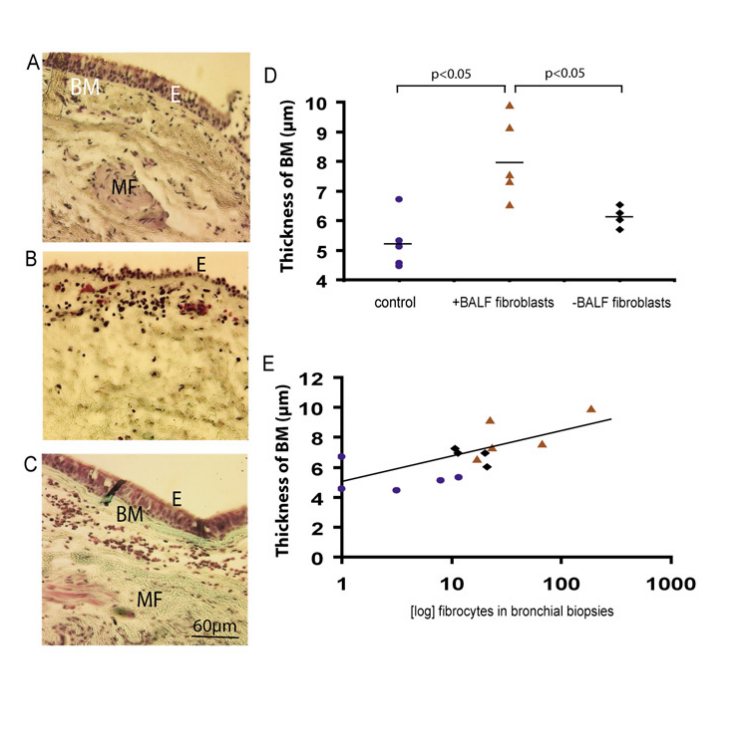
Basement membrane thickness in patients with mild asthma and controls. Bronchial biopsies from controls (n = 5) (A), patients with mild asthma with (+) BALF fibroblasts (n = 5) (B) and patients with mild asthma without (-) BALF fibroblasts (n = 4) (C) were stained with Gomoris trichrome staining for basic morphological characterization. Measurement of basement membrane thickness was performed as described in the Method section and is presented in μm (D). Number of fibrocytes in area A+B were counted and correlated (r = 0.711) to thickness of basement membrane (μm) (E). Values are presented as means ± SEM. Spearman coefficient of rank correlation was used to calculate the degree of association between basement membrane thickness and number of fibrocytes. (▲ patients with BALF fibroblasts, ▪ patients without BALF fibroblasts, ● controls). *Definition of abbreviations*: BM = Basement membrane, E = epithelium, MF = Muscle fibers.

The basement membrane thickness was measured in controls (Figure 5A), patients with asthma with BALF fibroblasts (Figure 5B), and patients without BALF fibroblasts (Figure 5C). The basement membrane was significantly thicker in patients with asthma with BALF fibroblast (7.95 ± 0.98 μm) when compared to the other patient group and controls (6.12 ± 0.18 μm and 5.23 ± 0.4 μm respectively, p < 0.05) (Figure 5D). A higher number of cells detected by hematoxylin staining beneath the basement membrane when compared to controls were characteristic for both groups of patients. Moreover, our data indicate a correlation between thickness of basement membrane and the presence of fibrocytes in the tissue of controls and patients with asthma (r = 0.711) (Figure 5E).

### BALF fibroblasts display fibrocyte characteristics

**Figure 6:**
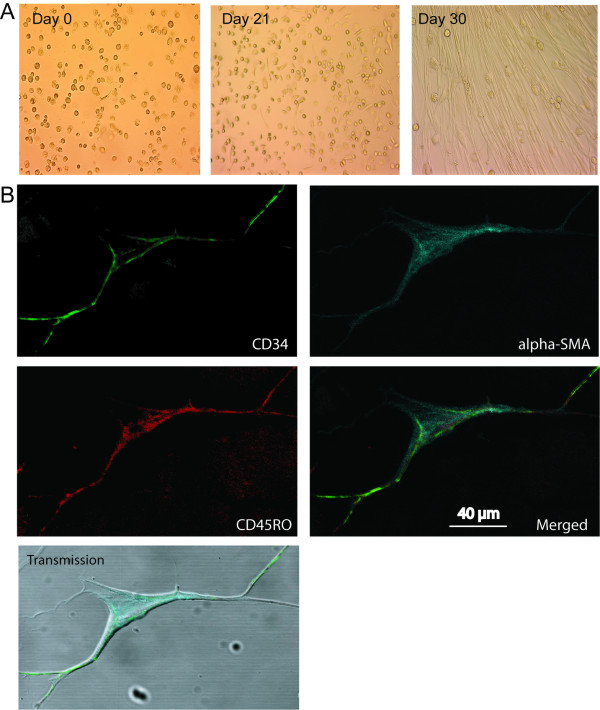
BALF fibroblasts in patients with mild asthma express the fibrocyte markers CD34, CD45RO, and α-SMA. BALF fibroblasts were cultured as described in the method section and observed after approximately 30 days in culture (A). The BALF fibroblasts were fixed in 4% paraformaldehyde and stained with antibodies against CD34 (green), CD45RO (red) and α-SMA (blue) (B). These pictures were merged together with a transmission picture.

BALF fibroblasts displayed an elongated, spindle-like shape after 20–30 days in culture (Figure 6A). So far, the origin of cultured BALF fibroblasts from patients with mild asthma has not been studied in detail. To see whether the BALF fibroblasts displayed characteristics of fibrocytes, these cells were triple-stained for CD34, CD45RO, and α-SMA, and subjected to confocal microscopy. Again, insignificant levels of background staining were seen indicating specificity for the antibodies used (data not shown). The BALF fibroblasts did indeed express the fibrocyte markers CD34, CD45RO, and α-SMA, which suggest that these cells may be derived from fibrocytes (Figure 6B). The number of BALF fibroblasts in passage 3–6 expressing all three fibrocyte markers constituted one fifth of the total cell count. Double-positive cells expressing CD34/α-SMA or CD45RO/α-SMA constituted 11.7 % and 5% respectively (Table 1 - figure 7). Moreover, 41 % of the cells stained positive only for α-SMA.

**Table 1:**
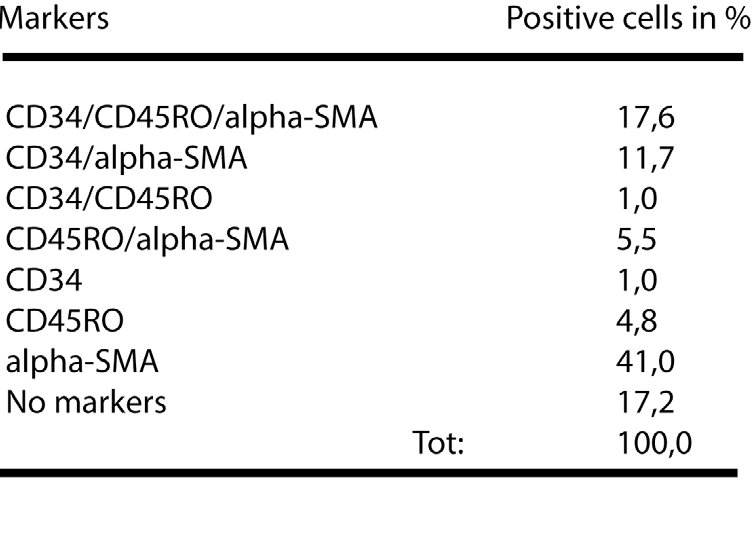
Fibrocyte markers expressed in the BALF fibroblasts. This table displays the number of positive cells in passage 3–6 from BALF fibroblast cultures from patients with mild asthma (n = 5) stained for the different fibrocyte markers used in the study.

Inflammatory cells such as eosinophils are suggested to be important in mediating the airway inflammation in patients with asthma and in vivo models [1,20]. Increased levels of BALF eosinophils have been reported in patients with BALF fibroblasts, which suggests an active inflammatory link to the presence of these fibroblasts [21]. However, we did not observe any correlation between the number of BALF eosinophils and fibrocytes in lung tissue from patients with mild asthma in this study (data not shown).

## Discussion

This is the first study to report an increased number of fibrocytes expressing CD34/CD45RO/α-SMA/procollagen I, and a localization of these cells to areas close to the basement membrane in steroid-naive patients with mild asthma when compared to controls. The inclusion of α-SMA coexpression to the other fibrocyte markers may indicate a possible link between circulating progenitor cells and their potential differentiation into α-SMA expressing myofibroblasts in these patients. It should be noted that the patients included in this study is very homogenous since they did not show any distinct clinical patterns except for five patients that had perennial allergy and were sensitive to cat.

Fibrocyte levels correlated to the thickness of the basement membrane proposing that these cells may participate in airway wall remodeling. The relative high levels of fibrocytes expressing α-SMA seen in patients with increased basement membrane thickness may indicate a more differentiated fibrocyte phenotype that is similar to the myofibroblast. In contrast to our findings, Schmidt and coworkers reported higher levels of collagen I expressing cells than α-SMA positive cells in ovalbumin treated mice and patients with allergen-provoked asthma [22]. Apart from the different study models used, these differences may be caused by alteration in cell differentiation which is difficult to monitor for fibrocytes in particular. Importantly, the analysis of prolyl-4-hydroxylase in our study indicates an active collagen production in the reported fibrocytes.

To our knowledge, this is the first study to report a correlation between tissue fibrocytes and increased thickness of basement membrane. Thickening of the lamina reticularis has been reported in the airways of children with severe asthma and has been shown to correlate to airway hyperresponsiveness, as well as an increased number of fibroblasts and myofibroblast in this area [23-25]. Apart from the differentiation of fibrocytes into myofibroblasts-like cells, additional sources of myofibroblasts, that may contribute to the enhanced extracellular matrix deposition during pathological conditions include resident myofibroblasts, differentiation of bronchial muscle cells and epithelial mesenchymal transition [26,27].

Molecules secreted by airway epithelium and surrounding inflammatory cells may explain the observed difference in numbers of fibrocytes in area A compared to area B and C. Factors that have previously been shown to be involved in the recruitment of fibrocytes to the fibrotic tissue include IL-1β, ED-A fibronectin, stromal-derived factor 1 (SDF1/CXCL12), granulocyte macrophage stimulating-factor (GM-CSF). Hence, the differentiated levels of fibrocytes and basement membrane thickness seen in this study may be a result of an altered local environment in the lungs of the two sub-groups of patients with mild asthma. Future screening of fibrocyte recruiting molecules in the BALF from these patients could provide a better understanding of the differences observed.

The outgrowth of fibroblasts in BALF from a sub-group of patients with mild asthma indicate a possible origin from circulating fibroblast progenitor cells, due to their relative high expression of fibrocyte markers CD34, CD45RO and α-SMA. Since the airway lumen has low levels of extracellular matrix, which is a prerequisite for survival of mature fibroblast, it is very likely that the BALF fibroblasts are derived from circulating fibrocytes rather than the residing tissue fibroblast pool. These lumen-derived cells were seen to correlate to the number of tissue fibrocytes and basement membrane thickness, indicating a possible link to the pathological changes in these patients.

When human fibrocytes are cultured in vitro, a decrease in the expression of CD34 and CD45RO occurs in parallel with an increased expression of α-SMA. This may confirm why one third of the original fibrocytes stained positive for α-SMA after three weeks. Similarly, approximately 50 % of the cells were stained positive only for α-SMA in passage 3–6 (Table 1). In the same passages, triple positive cells constituted approximately a fifth of the total cell count and double-positive for CD34/α-SMA or CD45RO/α-SMA constituted 10 % and 5 % respectively. This allow us to speculate that a homogenous population of CD34/CD45RO expressing fibrocytes in the BALF may have been the starting population, which has gradually differentiated into α-SMA expressing myofibroblast-like cells. Future studies, including detailed analysis of recovered cells expressing fibrocyte specific markers in BALF, may provide a clearer future view into this field.

BALF fibroblasts have previously been characterized as motile cells with a relatively high production of extracellular matrix components, including collagen and proteoglycans, accompanied by elevated levels of BALF eosinophils. In the present study, we did not observe any correlation of neither BALF nor tissue eosinophils to the number of fibrocytes in tissue. A possible explanation is that BALF eosinophils affect the differentiation of fibrocytes into myofibroblast-like cells due to their capacity of secreting TGF-β, rather than the recruitment of these cells to the lumen. In addition, we have preliminary data indicating a correlation between BALF neutrophils and tissue fibrocytes, suggesting an alternative inflammatory link to the recruitment of fibrocytes to the tissue (unpublished observation).

An important question remains to be answered: Do fibrocytes differentiate into myofibroblasts, or do they stimulate differentiation and proliferation of other progenitor cells or resident mesenchymal cells within the tissue?

In conclusion, the novel findings that cells from BALF in patients with mild asthma express fibrocyte specific proteins and the localization in tissue of these cells in close proximity to a thicker basement membrane emphasize the importance of these cells in the early stages of remodeling and inflammation. Further exploration in patients with severe asthma would be of paramount interest. The observation that patients with BALF fibroblasts with fibrocyte characteristics are accompanied by a thicker basement membrane suggests a specific pathological process for these patients when compared to patients lacking these cells. In the future, modulation of the recruitment and subsequent differentiation of fibrocytes may therefore be of great therapeutic importance in regulating airway wall remodeling in asthma.

## Conclusion

In conclusion, we hereby show that cells from BALF in patients with mild asthma express fibrocyte specific proteins and that these are localized in tissue in close proximity to a thicker basement membrane. These data, together with recent fibrocyte findings, emphasize the importance of these cells in airway remodeling in patients with mild asthma.

## Competing interests

The authors have declared that they have no financial or competing interest in the publication of this manuscript.

## Authors' contributions

KN: Participated in planning of the study, performed all confocal microscopy studies and have been involved in drafting the manuscript and performed all technical studies.

KL: Participated in the planning of the study, handled all cell work and drafted the main part of the abstract.

AHN: Participated and was involved the histology part of the study.

AM: Participated in the planning of the work.

LB: Clinician who participated in the planning of the study and recruited/collected all patient material.

GWT: Initiator of the study participated in the planning of the study, founder of the study.
